# The association between emotion malleability beliefs and severe psychological distress stratified by sex, age, and presence of any psychiatric disorders

**DOI:** 10.3389/fpsyg.2023.1251945

**Published:** 2023-10-10

**Authors:** Yohei Sasaki, Ryo Okubo, Kazuyoshi Takeda, Satoru Ikezawa, Takahiro Tabuchi, Kentaro Shirotsuki

**Affiliations:** ^1^Department of Human Sciences, Faculty of Human Sciences, Musashino University, Koto-ku, Japan; ^2^Department of Psychiatry and Neurology, National Hospital Organization Obihiro Hospital, Hokkaido, Japan; ^3^Department of Clinical Data Science, Clinical Research and Education Promotion Division, National Center of Neurology and Psychiatry, Tokyo, Japan; ^4^Graduate School of Arts and Sciences, The University of Tokyo, Tokyo, Japan; ^5^Cancer Control Center, Osaka International Cancer Institute, Osaka, Japan

**Keywords:** emotion regulation, emotion malleability beliefs, mindset, psychological distress, emotion belief

## Abstract

**Background:**

Recent studies have shown an association between psychological distress and emotion malleability beliefs, meaning mindsets about whether one’s emotions are fixed or changeable. However, most studies have not examined the association between these beliefs and sociodemographic factors.

**Methods:**

A nationwide cross-sectional Internet survey of residents of Japan aged 15–79 years was conducted using sampling weights for national estimates to investigate the association between emotion malleability beliefs and sociodemographic factors and between fixed beliefs and severe psychological distress (SPD). SPD was defined as a Kessler 6 Scale score of ≥13. Adjusted odds ratios for SPD were calculated considering potential confounders. Further analyses were stratified by sex, age and presence of any psychiatric disorder.

**Results:**

The analysis included 23,142 participants (female, 48.64%). Fixed beliefs were associated with female sex, age < 45 years, and presence of psychiatric disorders. These beliefs were associated with SPD, and additional analysis showed stronger associations with SPD among female respondents, respondents aged 45–59 years, and those aged ≥60 years.

**Conclusion:**

Results indicate that female sex, age < 45 years, and current mental disorders were associated with fixed emotion malleability beliefs. Associations between fixed emotion malleability beliefs and SPD were particularly strong among female respondents and people aged ≥45 years compared with the general population. Our study extends the association between emotion malleability beliefs and psychological health to the general population. Future studies should explore mechanisms underlying individual differences in emotion beliefs.

## 1. Introduction

Individuals’ belief systems, also known as implicit theories or mindsets (Dweck and Leggett, 1988), have been widely examined with respect to mental health. Specifically, there is growing interest in research on emotion malleability beliefs in relation to emotional problems (Kneeland et al., 2016; Burnette et al., 2020; Reffi et al., 2020; Schroder, 2021). People with more malleable beliefs about emotion show a positive correlation with positive emotions and well-being and a negative correlation with negative emotions and depressive symptoms (Tamir et al., 2007; King and dela Rosa, 2019). A recent meta-analysis demonstrated a negative association between fixed (less malleable) emotion beliefs and mental health. Moreover, previous studies suggest that individuals holding these beliefs frequently use maladaptive emotion regulation strategies (King and dela Rosa, 2019; Ortner and Pennekamp, 2020; Schell et al., 2023), which are associated with the development, persistence, and exacerbation of emotional disorders (Kneeland et al., 2016; Schroder, 2021).

Emotion dysregulation is at the core of trans-diagnostic mechanisms of emotional disorders (Gross, 2013; Sloan et al., 2017). Whether individuals believe emotions to be malleable or fixed affects their emotion regulation strategies and their experience of emotions, which is associated with psychopathologies such as depression and anxiety (for reviews, see Kneeland et al., 2016; Schroder, 2021). People who believe that emotions can be changed are more likely to engage in adaptive emotion regulation, such as cognitive reappraisal, in which they think of alternative interpretations of a situation (Tamir et al., 2007). On the other hand, people believe that emotions are fixed are more likely to be disengaged from regulation of their own emotions, and consequently to use maladaptive emotion regulation strategies such as avoidance and rumination (De Castella et al., 2018; Kneeland and Dovidio, 2020). These associations between fixed beliefs and psychological health have been observed in clinical research. In patients with social anxiety disorder, fixed beliefs independently explain variance in stress, trait anxiety, negative affect, and self-esteem, even after adjusting for the severity of social anxiety symptoms (De Castella et al., 2014). In patients with depression, fixed beliefs indirectly influence depressive symptoms via experiential avoidance (Sung et al., 2020). Thus, emotion malleability beliefs have been shown to have the potential to advance our understanding of the psychopathology of emotional disorders.

Although studies of emotion malleability beliefs should account for factors such as sex, age, and socioeconomic status, most have not. Some studies indicate that females are less likely to believe that they can regulate their own emotions (Nolen-Hoeksema and Jackson, 2001), whereas others found no gender differences (Tamir et al., 2007; De Castella et al., 2013, 2018). Such inconsistencies might be due to small sample sizes and lack of diversity among study participants. Most studies to date have included fewer than 1,000 participants, and have been conducted with young, Caucasian populations (Burnette et al., 2020). In addition, people who believe emotions are malleable tend to become stronger from early adulthood (18–25 years) to post-25 years old, although the difference was not statistically significant in comparison with adolescents aged 12–17 years (Burnette et al., 2020). Moreover, older people utilize maladaptive emotion regulation strategies less frequently than younger people (Schirda et al., 2016). These age differences in emotion regulation strategies might mean that older people have malleable beliefs about emotions than younger people. Similarly, differences in associations by race and the presence of psychiatric disorders have been examined, but no significant differences were found. Furthermore, the relationship between these beliefs and psychological health in people who have physical illnesses has not been clarified. The prevalence of depression is higher in people with physical illnesses such as hypertension, diabetes, and chronic pain compared with the general population (Gavard et al., 1993; Miller and Cano, 2009; Roy and Lloyd, 2012; Michal et al., 2013; Rayner et al., 2016). Thus, people with these illnesses may have some unique relationship between emotion malleability beliefs and psychological health. To our knowledge, no study has yet examined differences in these beliefs with respect to these factors in the general population.

Our study aimed to examine how emotion malleability beliefs vary by sex, age, health conditions and other socioeconomic factors and how these effect modifiers vary the strength of the association between these beliefs and psychological distress in a general population sample. This study is expected to provide some important findings. First, this study would reveal the relationship between emotion malleability beliefs and these factors. Empirical studies have shown that people who believe emotion is less malleable seek less support from their peers (Tamir et al., 2007) and they prefer medication to psychotherapy (Schroder et al., 2015). Thus, clarifying these associations would enable the dissemination of persuasive messages for improved mental health tailored to each group. Second, identifying a population with fixed beliefs in this study is expected to provide important data to advance the literature on emotion malleability beliefs. We expected that people with fixed beliefs would have higher rates of severe psychological distress than people with malleable beliefs. Therefore, we conducted an exploratory analysis without a specific hypothesis to examine the following research question: Do the associations between these beliefs and psychological distress differ according to the presence of various factors such as health status?

## 2. Methods

### 2.1. Study design and participants

This study was a secondary analysis of data from the second wave of the Japan “COVID-19 and Society” Internet Survey (JACSIS; Yoshioka et al., 2021). The JACSIS study complied with ethical standards of the relevant national and institutional committees on human experimentation and the 1975 Declaration of Helsinki (as revised in 2008). The protocol was approved by the Research Ethics Committee of the Osaka International Cancer Research Institute (June 19, 2020; approval number 20084), and the survey allows for secondary use of the data. The protocol for the present study was reviewed and approved by the Research Ethics Committee of the [Anonymised for review] (June 11, 2022; approval number 2021-31-02).

In September 2020, the JACSIS study conducted a nationwide baseline survey of 28,000 male and female respondents aged 15–79 years who were selected randomly from 224,389 panelists registered with a large Internet survey company (Rakuten Insight, Inc., Tokyo, Japan).^1^ In February 2021, a follow-up survey was conducted in which 24,059 of the 28,000 baseline survey participants took part. In addition, 1,941 participants aged 15–79 years were newly recruited, without stratification by age, sex, or prefecture of residence, giving a total of 26,000 participants. “E-points” (points used for Internet shopping or cash exchange) were offered to encourage participation. Although the survey agency declined to disclose the exact value of the e-points, one E-point is considered equivalent to approximately 100 yen (approximately US$1 at the time of the survey). The study was a cross-sectional study that used data obtained from this follow-up survey, in which participants responded to a scale to measure their beliefs about emotion malleability.

### 2.2. Measures

#### 2.2.1. Emotion malleability beliefs

The Japanese version of the Personal Implicit Beliefs About Emotions scale (De Castella et al., 2013) assessed emotion malleability beliefs. This questionnaire consists of four items, two of which employ reversed items (“I can learn to control my emotions,” “If I want to, I can change the emotions that I have”). Each item is rated on a 5-point scale (1 = strongly disagree, 5 = strongly agree). A higher total score means that they strongly believe in a lack of control over their emotions.

We developed the Japanese version of this scale based on a report by a working group of the International Society for Pharmacoeconomics and Outcomes Research (Wild et al., 2005). This scale was independently translated from English to Japanese by a psychologist and a bilingual Japanese-English psychiatrist, both of whom integrated the translations through discussion. In addition, another bilingual Japanese-English psychiatrist compared the original version with the Japanese version and made corrections to the translation. The preliminary Japanese version was then back translated into English by a bilingual individual employed at a translation company who was unfamiliar with the original version. The author of the original version reviewed the back-translation and confirmed the equivalence of its semantic content with that of the original version.

De Castella et al. (2013) did not report the factor structure of the Personal Implicit Beliefs about Emotion Scale. In our analyses below, we used the total score of this scale as in previous studies (De Castella et al., 2013, 2014). In the present study, internal reliability was demonstrated based on Cronbach’s α of 0.59 and McDonald’s ω of 0.66, which were slightly lower compared with the Cronbach’s alpha of 0.73 reported previously (De Castella et al., 2018).

#### 2.2.2. Severe psychological distress

The Japanese version of The Kessler Psychological Distress Scale, a widely used measure of psychological distress in the general population, was used to assess psychological distress (Furukawa et al., 2003). For this study, severe psychological distress (SPD) was defined as a total score of 13 or higher. A score of 13 was a cutoff to screen for severe mood disorder or any anxiety disorder (Furukawa et al., 2003; Kessler et al., 2003). The present study showed good internal consistency (*α* = 0.93; *ω* = 0.94).

#### 2.2.3. Sociodemographic factors

The following sociodemographic factors were considered: sex, age (15–29, 30–44, 45–59, 60–79 years), income (low, <JPY2.5 million/US$25,000/£16,667; intermediate, JPY2.5–JPY4.3 million/US$25,000–US$43,000/£16,667–£28,667; high, <JPY4.3 million/<US$43,000/<£28,667; unknown/declined to answer), marital status (unmarried, married, and widowed/separated), education, working status (working, schooling, homemaker, not working), history of tobacco use, history of alcohol use, and presence of current illness (hypertension, diabetes mellitus, asthma or chronic obstructive pulmonary disease, cardiovascular disease, cerebrovascular disease, cancer, chronic pain, psychiatric disorders).

### 2.3. Statistical methods

First, an inverse probability weighting (IPW) method was used to correct for differences between Internet survey respondents and the general population. By logistic regression analysis, propensity scores (weights) were calculated using sex, age, and socioeconomic factors to correct for differences between respondents to the current Internet survey and respondents to the 2019 National Survey on Living Standards, which is representative of the Japanese population. Details of this calculation are described in our previous studies (Okubo et al., 2021a,b). Second, an individual’s malleable beliefs are described on a spectrum, with malleable beliefs at one pole and fixed beliefs at the other pole (Dweck and Leggett, 1988); therefore, we trichotomized participants’ beliefs as malleable (T1: total score from 4 to 10), neither (T2: total score from 11 to 12), or fixed (T3: total score from 13 to 20 scores), where high signifies a strong belief in emotion malleability. Implicit theories research explains belief effects by contrasting individuals who do believe in emotion malleability with those who do not (Dweck et al., 1995; Tamir et al., 2007). Finally, the association between emotion beliefs and SPD was clarified by calculating adjusted odds ratios (aORs) and 95% confidence intervals (CIs) for SPD by logistic regression analysis with IPW based on propensity scores. In these analyses, all variables noted above were entered into the model as independent variables. All analyses were performed using R software version 4.0.3.

## 3. Results

Of the 26,000 study participants, 2,858 were excluded for satisficing or straight-lining or discrepant survey responses (for example, respondents who chose inappropriate responses to the instruction “Please choose the second from the bottom of the following options.” or respondents who answered “currently have this condition and receiving treatment” or “currently have this condition but not receiving treatment” (as opposed to “never in the past” or “not now, but had in the past”) to all 16 questions regarding the following comorbidities). Thus, the analysis included data from 23,142 (89.0%) participants. The sociodemographic characteristics of the participants are shown in Table 1. Among 23,412 participants, the median total score for implicit beliefs about emotions was divided into tertiles, namely, a malleable group (T1), an neither group (T2), and a fixed group (T3) with median total scores of 16, 12, and 10, respectively. The high group had 8,123 participants (35.1%), the middle group 11,465 participants (49.5%), and the fixed group 3,554 participants (15.4%).

**Table 1:** Participant characteristics (before and after propensity score weighting).

Variables	After weighting		Before weighting	
	*n*	%	*n*	%
Sex				
Male	11,766	50.8	11,766	50.8
Female	11,376	49.2	11,376	49.2
Age (years)				
15–29	3,448	14.9	3,476	15.0
30–44	5,255	22.7	5,330	23.0
45–59	6,711	29.0	6,614	28.6
≥60	7,728	33.4	7,722	33.4
Income (million JPY/year)				
Less than 1	841	3.6	898	3.9
1 to less than 6	10,699	46.2	10,310	44.6
6 to less than 12	5,433	23.5	5,938	25.7
12 or more	952	4.1	1,308	5.7
No response/unknown	5,217	22.5	4,688	20.3
Marital status				
Married	13,669	59.1	12,560	54.3
Never married	4,876	21.1	5,770	24.9
Widowed/divorced	4,596	19.9	4,812	20.8
Education				
Junior high school graduate	4,114	17.8	3,366	14.5
High school graduate	9,739	42.1	6,012	26.0
Junior college graduate	3,366	14.5	4,247	18.4
Bachelor’s degree	4,315	18.7	8,576	37.1
Master’s or doctoral degree	1,608	7.0	941	4.1
Living alone, yes	3,535	15.3	4,504	19.5
Working status				
Working	11,955	51.7	14,315	61.9
Schooling	2,246	9.7	1,275	5.5
Homemaker	4,358	18.8	3,800	16.4
Not working	4,334	18.7	3,752	16.2
Smoking status				
Never	11,955	51.7	12,709	54.9
Ever	9,188	39.7	8,731	37.7
Current	1999	8.6	1702	7.4
Alcohol use				
Never	4,111	17.8	3,791	16.4
Ever	7,373	31.9	7,379	31.9
Current	11,658	50.4	11,972	51.7
Current medical history				
Hypertension	4,490	19.4	4,359	18.8
Diabetes mellitus	1,628	7.0	1,467	6.3
Asthma or COPD	1,153	5.0	959	4.1
Cardiovascular disease	627	2.7	464	2.0
Cerebrovascular disease	260	1.1	222	1.0
Cancer	442	1.9	435	1.9
Chronic pain	2,330	10.1	1939	8.4
Psychiatric disorders	1,463	6.3	1,256	5.4
Severe Psychological Distress (K6 ≥ 13)	2,741	11.8	2,570	11.1

COPD, chronic obstructive pulmonary disease; K6, Kessler Psychological Distress Scale.

Table 2 shows sociodemographic characteristics factors associated with emotion malleability beliefs. We found that female respondents had significantly fixed beliefs than male respondents (aOR = 1.31; 95% CI = 1.18–1.44, *p* < 0.001). Compared with people aged 45–59 years, those aged 29 years or younger (aOR = 1.24; 95% CI = 1.09–1.41, *p* < 0.001) and those aged 30–44 years (aOR = 1.16; 95% CI = 1.05–1.28, *p* < 0.01) had fixed beliefs, whereas those aged 60 years or older had malleable beliefs (aOR = 0.57; 95% CI = 0.51–0.65, *p* < 0.001). In terms of income, we found that compared with the group earning 1 million to 6 million yen, the group earning 6 million to 12 million yen had significantly fixed beliefs (aOR = 1.13; 95% CI = 1.02–1.24, *p* < 0.05), whereas the non-response group had significantly malleable beliefs (aOR = 0.85; 95% CI = 0.77–1.13, *p* < 0.01). As for marital status, never-married people had significantly fixed beliefs compared with married people (aOR = 1.13; 95% CI = 1.02–1.27, *p* < 0.05). Regarding educational background, compared with respondents with a bachelor’s degree, junior high school graduates (aOR = 1.20, 95% CI = 1.04–1.39, *p* < 0.05) and high school graduates (aOR = 1.15, 95% CI = 1.04–1.28, *p* < 0.01) had significantly fixed beliefs, whereas those with a master’s degree or above had significantly malleable beliefs (aOR = 0.22, 95% CI = 0.16–0.30, *p* < 0.001). Meanwhile, people living alone had significantly fixed beliefs than people living with others (aOR = 0.87, 95% CI = 0.77–0.98, *p* < 0.05). We found that emotion malleability beliefs were significantly fixed in ever-smokers (aOR = 1.20, 95% CI = 1.08–1.35, *p* < 0.001) and current smokers (aOR = 1.16, 95% CI = 1.04–1.30, *p* < 0.01) than in never-smokers. In terms of current medical history, people with hypertension (aOR = 1.19, 95% CI = 1.07–1.32, *p* < 0.001), diabetes (aOR = 1.21, 95% CI = 1.04–1.40, *p* < 0.01), chronic pain (aOR = 1.45, 95% CI = 1.29–1. 63, *p* < 0.001), or psychiatric disorders (aOR = 2.79, 95% CI = 2.46–3.16, *p* < 0.001) had significantly fixed beliefs, whereas people with cerebrovascular disease (aOR = 0.69, 95% CI = 0.48–0.98, *p* < 0.05) or cancer (aOR = 0.63, 95% CI = 0.44–1.82, *p* < 0.01) had significantly malleable beliefs.

**Table 2:** Association between sociodemographic characteristics and total scores of binarized (malleable + neither vs. ficed) emotion malleability beliefs.

Emotion malleability beliefs	Malleable + neither		Fixed			
Variables	*n*	%	*n*	%	aOR	95% CI
Sex						
Male	10,063	51.37	1,703	47.93	1.00	ref
Female	9,525	48.63	1,851	52.08	1.31^***^	(1.20–1.43)
Age (years)						
15–29	2,754	14.06	694	19.53	1.24^***^	(1.09–1.41)
30–44	4,276	21.83	980	27.56	1.16^**^	(1.05–1.28)
45–59	5,572	28.45	1,139	32.04	1.00	ref
≥60	6,986	35.67	742	20.87	0.57^***^	(0.51–0.65)
Income (million JPY/year)						
Less than 1	690	3.52	150	4.23	0.96	(0.79–1.17)
1 to less than 6	9,097	46.44	1,603	45.09	1.00	ref
6 to less than 12	4,484	22.89	950	26.73	1.13^*^	(1.02–1.24)
12 or more	824	4.21	128	3.59	0.82	(0.67–1.01)
No response/unknown	4,494	22.94	723	20.35	0.85^**^	(0.77–0.94)
Marital status						
Married	11,733	59.90	1,936	54.47	1.00	ref
Never married	3,995	20.40	881	24.79	1.13^*^	(1.01–1.27)
Widowed/divorced	3,859	19.70	737	20.75	0.99	(0.87–1.13)
Education						
Junior high school	3,401	17.36	713	20.08	1.20^*^	(1.04–1.39)
High school	8,152	41.62	1,586	44.63	1.15^**^	(1.04–1.28)
Junior college graduate	2,808	14.34	558	15.71	1.06	(0.93–1.21)
Bachelor’s degree	3,672	18.75	642	18.08	1.00	ref
Master’s or doctoral degree	1,555	7.94	54	1.51	0.22^***^	(0.16–0.30)
Living together (with one or more persons)	16,512	84.29	3,095	87.08	1.00	ref
Living alone	3,077	15.71	459	12.92	0.87^*^	(0.77–0.98)
Working status						
Working	10,139	51.76	1,816	51.11	1.02	(0.90–1.14)
Schooling	1,850	9.44	396	11.14	1.03	(0.83–1.29)
Homemaker	3,698	18.88	659	18.55	1.00	ref
Not working	3,790	19.35	544	15.31	1.16	(1.00–1.35)
Smoking status						
Never	10,139	51.76	1,816	51.11	1.00	ref
Ever	7,742	39.52	1,447	40.70	1.20^***^	(1.08–1.35)
Current	1,708	8.72	291	8.19	1.16^**^	(1.04–1.30)
Alcohol use						
Never	3,534	18.04	577	16.24	1.00	ref
Ever	6,100	31.14	1,272	35.80	1.02	(0.93–1.11)
Current	9,954	50.82	1,704	47.96	0.96	(0.83–1.11)
Current medical history						
Hypertension	3,817	19.49	674	18.95	1.19^***^	(1.07–1.32)
Diabetes mellitus	1,344	6.86	284	8.00	1.21^**^	(1.04–1.40)
Asthma or COPD	918	4.69	235	6.62	1.17	(0.99–1.38)
Cardiovascular disease	502	2.56	126	3.54	1.23	(0.96–1.57)
Cerebrovascular disease	212	1.08	48	1.34	0.69^*^	(0.48–0.98)
Cancer	372	1.90	70	1.97	0.63^**^	(0.46–0.84)
Chronic pain	1,835	9.37	495	13.93	1.45^***^	(1.29–1.63)
Psychiatric disorders	964	4.92	498	14.03	2.79^***^	(2.46–3.16)

COPD, chronic obstructive pulmonary disease; OR, odds ratio; CI, confidence interval. aORs were adjusted for age, sex, income, marital status, education, living together, working status, use of combustible cigarettes or heated tobacco products, use of alcohol, current medical history. These were calculated by comparing the low emotional malleability beliefs group with the high and low groups. ^***^p < 0.001, ^**^p < 0.01, ^*^p < 0.05 for each OR.

Table 3 shows the relationship between fixed malleability beliefs and risk of SPD. The aORs for the association between these beliefs and SPD were higher in the neither group (aOR = 2.48, 95% CI = 2.18–2.83, trend *p* < 0.001) and fixed group (aOR = 7.55, 95% CI = 6.56–8.68, trend *p* < 0.001) than in the malleable group. For relationships between fixed beliefs and SPD by sociodemographic characteristic factors, when compared with all participants, there was a strong association between fixed beliefs and SPD in the neither group (aOR = 3.69, 95% CI = 3.03–4.49, trend *p* < 0.001) and fixed group (aOR = 11.42, 95% CI = 9.27–14.08, trend *p* < 0.001) among females, the fixed group among those aged 45–59 years, and in the neither group (aOR = 8.81, 95% CI = 6.89–11.26, trend *p* < 0.001) and fixed group (aOR = 30.32, 95% CI = 14.56–64.05, trend *p* < 0.001) among those aged 60 years or older.

**Table 3:** Odds ratio and 95% confidence intervals for severe psychological distress according to the trisect of the total score on emotion malleability beliefs (lower score = lower malleability).

	Malleable	Neither	Fixed	Trend *p*
Total score				
Median (min-max) score	8 (4–10)	12 (11–12)	14 (13–20)	
Number of cases/controls	337/7786	1299/10165	1104/2450	
Age, sex-adjusted OR (95% CI)	1.00 (ref)	2.40 (2.12–2.72)	8.12 (7.11–9.27)	***
Multivariate aOR (95% CI)	1.00 (ref)	2.48 (2.18–2.83)	7.55 (6.56–8.68)	***
Male				
Median (min-max) score	8 (4–10)	13 (12–11)	12 (13–20)	
Number of cases/controls	194/3593	611/5664	448/1256	
Age-adjusted OR (95% CI)	1.00 (ref)	1.72 (1.45–2.04)	5.74 (4.77–6.90)	***
Multivariate aOR (95% CI)	1.00 (ref)	1.66 (1.39–1.99)	5.23 (4.30–6.34)	***
Female				
Median (min-max) score	8 (4–10)	11 (12–12)	14 (13–20)	
Number of cases/controls	143/4192	689/4501	657/1194	
Age-adjusted OR (95% CI)	1.00 (ref)	3.55 (2.94–4.29)	12.26 (10.08–14.92)	***
Multivariate aOR (95% CI)	1.00 (ref)	3.69 (3.03–4.49)	11.42 (9.27–14.08)	***
Age				
Age 15–29 years				
Median (min-max) score	9 (4–11)	12 (12–12)	14 (13–20)	
Number of cases/controls	122/711	91/231	563/1729	
Sex-adjusted OR (95% CI)	1.00 (ref)	0.80 (0.66–0.98)	3.71 (3.00–4.58)	***
Multivariate aOR (95% CI)	1.00 (ref)	0.91 (0.73–1.13)	3.55 (2.83–4.46)	***
Age 30–44 years				
Median (min-max) score	9 (4–11)	12 (12–12)	14 (13–20)	
Number of cases/controls	89/1280	74/376	709/2728	
Adjusted OR (95% CI)	1.00 (ref)	1.68 (1.38–2.04)	5.87 (4.77–7.22)	***
Multivariate aOR (95% CI)	1.00 (ref)	1.74 (1.41–2.14)	5.16 (4.14–6.43)	***
Age 45–59 years				
Median (min-max) score	8 (4–10)	12 (11–12)	14 (13–20)	
Number of cases/controls	98/2071	368/3035	361/778	
Adjusted OR (95% CI)	1.00 (ref)	2.56 (2.04–3.22)	9.72 (7.67–12.33)	***
Multivariate aOR (95% CI)	1.00 (ref)	2.33 (1.84–2.95)	8.81 (6.89–11.26)	***
Age ≥ 60 years				
Median (min-max) score	7 (4–9)	12 (10–12)	14 (13–20)	
Number of cases/controls	9/2246	107/2215	149/3001	
Adjusted OR (95% CI)	1.00 (ref)	12.59 (6.92–22.90)	28.68 (15.20–54.10)	***
Multivariate aOR (95% CI)	1.00 (ref)	13.08 (6.58–26.02)	30.32 (14.36–64.05)	***
Education				
Junior high school				
Median (min-max) score	8 (4–10)	12 (11–12)	14 (13–20)	
Number of cases/controls	52/1186	253/1910	252/461	
Age, −adjusted OR (95% CI)	1.00 (ref)	2.69 (1.97–3.66)	10.25 (7.42–14.16)	***
Multivariate aOR (95% CI)	1.00 (ref)	2.16 (1.54–3.03)	7.75 (5.41–11.11)	***
High school				
Median (min-max) score	8 (4–10)	12 (11–12)	14 (13–20)	
Number of cases/controls	139/2754	463/4797	463/1123	
Age, −adjusted OR (95% CI)	1.00 (ref)	1.55 (1.27–1.89)	6.74 (5.48–8.28)	***
Multivariate aOR (95% CI)	1.00 (ref)	1.46 (1.18–1.81)	5.92 (4.73–7.42)	***
Junior college graduate				
Median (min-max) score	8 (4–10)	12 (11–12)	14 (13–20)	
Number of cases/controls	44/1072	246/1445	165/394	
Age, −adjusted OR (95% CI)	1.00 (ref)	3.60 (2.58–5.02)	8.72 (6.11–12.45)	***
Multivariate aOR (95% CI)	1.00 (ref)	3.23 (2.24–4.64)	8.08 (5.47–11.92)	***
Bachelor’s degree				
Median (min-max) score	8 (4–10)	12 (11–12)	14 (13–20)	
Number of cases/controls	91/1615	239/1727	207/435	
Age, −adjusted OR (95% CI)	1.00 (ref)	2.03 (1.58–2.62)	6.96 (5.29–9.16)	***
Multivariate aOR (95% CI)	1.00 (ref)	1.91 (1.44–2.53)	5.38 (3.96–7.31)	***
Master’s or doctoral degree				
Median (min-max) score	7 (4–9)	12 (10–12)	14 (13–20)	
Number of cases/controls	3/747	83/511	39/226	
Age, −adjusted OR (95% CI)	1.00 (ref)	40.60 (12.54–131.45)	26.29 (7.63–90.56)	***
Multivariate aOR (95% CI)	1.00 (ref)	1.46 (1.18–1.81)	5.92 (4.73–7.42)	***
Without psychiatric disorders				
Median (min-max) score	8 (4–10)	12 (11–12)	14 (13–20)	
Number of cases/controls	296/7528	1023/9777	766/2289	
Age, −adjusted OR (95% CI)	1.00 (ref)	2.08 (1.82–2.38)	6.46 (5.58–7.47)	***
Multivariate aOR (95% CI)	1.00 (ref)	1.93 (1.67–2.23)	6.10 (5.21–7.13)	***
With psychiatric disorders				
Median (min-max) score	12 (4–12)	13 (13–13)	15 (14–20)	
Number of cases/controls	164/305	154/341	338/160	
Age, −adjusted OR (95% CI)	1.00 (ref)	2.28 (1.58–3.29)	5.83 (4.39–7.73)	***
Multivariate aOR (95% CI)	1.00 (ref)	2.46 (1.64–3.69)	7.57 (5.47–10.46)	***

OR, odds ratio; CI, confidence interval. aORs were adjusted for age, sex, income, marital status, education, living together, working status, use of combustible cigarettes or heated tobacco products, use of alcohol, current medical history. These were calculated by comparing the low emotional malleability beliefs group with the high and low groups. ***p < 0.001.

## 4. Discussion

This study aimed to examine differences in emotion malleability beliefs across sex, age, and socioeconomic factors and differences in the relative strength of the association between these beliefs and SPD across effect modifiers, such as sex and age, using a large general population sample. Logistic regression analysis revealed a significant association between fixed beliefs and female sex, younger age, lower education, unmarried status, nonemployment, smoking, and the presence of hypertension, diabetes, chronic pain, or psychiatric disorders. In addition, fixed beliefs were a risk factor for SPD, with the association stronger for female respondents and respondents aged 45 years or older than for all participants.

The proportion of respondents with high emotion malleability beliefs increased with age. This result is consistent with previous research showing that older adults exhibit more mature emotional processing and regulation strategies than younger adults (Lawton, 2001; Blanchard-Fields, 2007; Urry and Gross, 2010). Older adults are characterized by their ability to pay attention to positive aspects and to construct better evaluations when confronted with negative information (Charles and Luong, 2013). When compared with younger adults, older adults are reported to utilize more adaptive emotion regulation strategies, such as acceptance, and fewer maladaptive emotion regulation strategies during anxiety- and sadness-provoking situations (Schirda et al., 2016).

We found that female respondents had fixed beliefs than male. Although several studies have reported no sex differences in emotion malleability beliefs (Tamir et al., 2007; De Castella et al., 2013, 2018), all of these studies had small sample sizes (*n* = 101–437) and did not adjust for confounders such as educational background, income, and current illness. This is the first study to examine differences in the distribution of emotion malleability beliefs using a large sample (>10,000 people). Interestingly, the association between fixed beliefs and SPD was more than twice as strong in female respondents than in male respondents. This difference can be explained by sex differences in emotion regulation strategies that are strongly related to emotion malleability beliefs. Female tend to focus more attention on their emotions than do male and they engage in excessive rumination to understand their feelings and the causes of their emotions, resulting in emotion regulation failure (Nolen-Hoeksema, 2012). Meta-analyses found that female ruminate more frequently than male (Rood et al., 2009; Johnson and Whisman, 2013). Failure to regulate emotions reinforces the belief that emotions are uncontrollable. In addition, these differences in these beliefs between male and female may be partially explained by the influence of psychosocial development on sex differences. According to gender socialization theory, girls of a certain generation are expected to internalize negative emotions and display more empathy and sympathy than boys of a certain generation (Brody and Hall, 2008; Zahn-Waxler et al., 2008). Female may be affected by cultural pressures that promote the internalization of negative emotions and may develop the belief that controlling emotions is difficult.

Our finding that people with any psychiatric disorder have fixed beliefs than those without is consistent with previous studies showing fixed beliefs in patients with social anxiety disorder (De Castella et al., 2014) and depressive disorder (Sung et al., 2020) compared with healthy individuals. Our findings support the theory that people who believe that emotions are uncontrollable are less motivated to effectively regulate negative emotions and become increasingly symptomatic (Kneeland et al., 2016; Schroder, 2021). Studying individual differences in how people respond to their own emotions can help clarify the mechanisms underlying depression and anxiety disorders and elaborate psychological interventions (Campbell-Sills et al., 2007).

This study is the first to examine the association between physical illness and emotion malleability beliefs. Our results showed that people with hypertension, diabetes, and chronic pain had fixed beliefs. People with these illnesses have a high prevalence of depression. More specifically, the prevalence of depression is 21.3% in people with hypertension (Michal et al., 2013) and 30% in people with chronic pain (Miller and Cano, 2009; Rayner et al., 2016); and people with diabetes have a prevalence of depression 2–3 times that in the general adult population (Gavard et al., 1993; Roy and Lloyd, 2012). Emotion beliefs can partially explain the differences in the prevalence of depression between people with physical illness and healthy individuals. In addition, people with fixed emotion malleability beliefs tend to prefer medication over psychotherapy (Schroder et al., 2015). Psychotherapy is reported to be effective in improving comorbid depression (Li et al., 2017, 2021; Ólason et al., 2018). Therefore, clinicians might first need to motivate their patients to believe in the malleability of their emotions to enhance their motivation for treatment before providing psychological intervention.

Interestingly, our results showed that cancer patients have high emotion malleability beliefs. Some researchers have proposed that more than two-thirds of adults cancer patients do not experience clinical anxiety or mood disorders (^24,30^ Linden et al., 2015) because most people can effectively regulate their emotions throughout the disease course (Kangas and Gross, 2020). Future research should investigate changes in emotion malleability beliefs throughout the course of cancer to gain a more detailed understanding of emotion regulation processes.

The association between fixed beliefs and SPD was stronger in older adults than in younger adults. One explanation is that mediators of the association between fixed beliefs and SPD differ between younger and older adults. For example, people with fixed beliefs are less likely to receive support from new friends (Tamir et al., 2007). Fifty-five percent of older adults experience moderate or greater loneliness and social isolation, which significantly reduces their mental health (Musich et al., 2015; Erzen and Çikrikci, 2018). Future research should examine factors such as loneliness that can specifically mediate these beliefs and SPD in older adults. Development of an education or intervention program that focuses on these mediators might be needed as well.

This study has several limitations that should be considered. First, the sample was collected through an Internet survey, which does not fully reflect the demographic distribution of the general population. Although we conducted weighted sampling utilizing nationally representative data to adjust for potential bias, it is possible that only people with a high affinity for Internet use participated in the survey. Second, this cross-sectional study cannot deny the reverse causation. For example, severe depression or anxiety might cause people to believe that their emotions are fixed. Finally, recent studies have shown that beliefs about emotions are not only malleability but also include beliefs such as useful and unfriendly (Ford and Gross, 2019; Veilleux et al., 2021). The present study cannot exclude the possibility that other emotion beliefs may have a more important relationship with psychological distress.

In conclusion, we found that the beliefs that individuals hold about emotions are fixed was associated with female sex, younger age, lower educated, unmarried status, nonemployment, smoking, and hypertension, diabetes, chronic pain, and psychiatric disorders. Furthermore, the association between fixed beliefs and SPD was stronger in female respondents and the elderly. These results can be explained by differences in factors such as loneliness and emotion regulation strategies, which might mediate the association between emotion malleability beliefs and SPD. Future studies are needed to compare the strength of the association between emotion malleability beliefs and emotion regulation strategies, such as rumination, using simultaneous multi-population analyses to partially explain the 2–3 times higher risk of major depressive disorder in female compared with men (Van de Velde et al., 2010; Breslau et al., 2017). In addition, it would be beneficial to provide group education on emotion malleability beliefs and psychological distress in educational settings utilized by many young people with fixed beliefs. An intervention for middle school students was conducted with the aim of improving mental health by modifying these beliefs (Smith et al., 2018), and such intervention programs targeting emotion beliefs in young people should be further developed. Furthermore, people with fixed beliefs tend to be less motivated to engage in psychotherapy (Schroder et al., 2015), so it is important to measure emotion malleability beliefs when administering psychotherapy. Psychoeducation about the controllability of emotions prior to psychotherapy is a potentially beneficial clinical strategy for motivating patients with fixed beliefs to engage in evoke strong emotion techniques, such as exposure (Kneeland et al., 2016). Our research supports the possibility that the study of emotion malleability beliefs can contribute to the development of more tailored strategies and interventions to improve psychological health.

## Data availability statement

The data analyzed in this study is subject to the following licenses/restrictions: all anonymized individual participant data reported in this paper are available for interested researchers who send a request for data sharing, along with a synopsis of the secondary analysis plan paper to the RO. Requests to access these datasets should be directed to RO, rokubo0425@gmail.com.

## Ethics statement

The studies involving humans were approved by the Research Ethics Committee of the Osaka International Cancer Research Institute and the Research Ethics Committee of the Musashino University. The studies were conducted in accordance with the local legislation and institutional requirements. Written informed consent for participation was not required from the participants or the participants’ legal guardians/next of kin because in accordance with national legislation and institutional requirements, written informed consent was not required for this study.

## Author contributions

YS and RO performed the data analysis. YS, RO, and KS drafted the manuscript. RO and TT designed the survey and collected the data. All authors contributed to the manuscript and agreed to submit it for publication.

## Footnotes

^1^ https://in.m.aipsurveys.com
